# Oral Candida in Patients with Fixed Orthodontic Appliance: In Vitro Combination Therapy

**DOI:** 10.1155/2017/1802875

**Published:** 2017-06-08

**Authors:** Wisam Alhamadi, Rafal J. Al-Saigh, Nebras N. Al-Dabagh, Hussam W. Al-Humadi

**Affiliations:** ^1^Department of Orthodontics, Dentistry College, University of Babylon, Babylon, Iraq; ^2^Department of Clinical & Laboratory Sciences, Pharmacy College, University of Babylon, Babylon, Iraq; ^3^Department of Basic Sciences, Dentistry College, University of Babylon, Babylon, Iraq; ^4^Department of Pharmacology & Toxicology, Pharmacy College, University of Babylon, Babylon, Iraq

## Abstract

**Background:**

Fixed orthodontic appliance (FOA) increases the cariogenic microorganisms of mouth including candida. The aim was to evaluate the pharmacodynamic effects of some antibacterial drugs in combination with most applicable antifungal agents on candida isolated from patients with FOA.

**Methods:**

Three antifungal agents (amphotericin B (AMB), ketoconazole (KET), and itraconazole (ITZ)) and three antibacterial drugs (ciprofloxacin (CIP), doxycycline (DOX), and metronidazole (MET)) with serial concentrations have been used and microdilution broth method has been done for single and combination therapy, then fungal growth was assessed spectrophotometrically, and the combinations were evaluated by bliss independent analysis.

**Results:**

According to bliss independent interaction, the synergistic interactions depended on Δ*E* values that showed the best for CIP was with AMB (Δ*E* = 55.14) followed with KET (Δ*E* = 41.23) and lastly ITR (Δ*E* = 39.67) at CIP = 150 mg/L. DOX was optimal with KET (Δ*E* = 42.11) followed with AMB (Δ*E* = 40.77) and the lowest with ITR (Δ*E* = 9.12) at DOX = 75 mg/L. MET is the best with AMB (Δ*E* = 40.95) and then with ITR (Δ*E* = 35.45) and finally KET (Δ*E* = 15.15) at MET 200 mg/L. Moreover, usage of higher concentrations of antibacterial agents revealed inhibitory effects.

**Conclusion:**

This study uncovers the optimum antibiotic combination therapy against cariogenic candida with FOA by usage of low therapeutic concentrations.

## 1. Introduction

The orthodontic treatment of malocclusions includes the conversion of mechanical energy generated by fixed orthodontic appliance (FOA) forces to biological reaction in teeth and supporting tissues as gingival inflammation and retraction in response to tooth movement [1] which are considered as a low risk and noninvasive orthodontic procedures [2, 3]. The microbial flora of the mouth is usually a mixture of microorganisms and may consist of more than 200 species [4]. The acid-producing bacteria usually colonize on the tooth surface and surrounding FOA or on orthodontic brackets leading to enamel demineralization [4, 5]. Nonetheless, the orthodontic brackets have also effect on good oral hygiene leading to plaque accumulation and increase in cariogenic microorganisms in saliva like candida and dental plaque of patients [5, 6] as the presence of oral braces increases the frequency of colonization by* Candida *species [6–8].

*Candida* species are frequently found in the oral cavity, with a percentage of up to 60% in young adults [8, 9].* Candida albicans* is the prevalent species; however, other species such as* C. dubliniensis, C. parapsilosis C. tropicalis*,* C. krusei,* and* C. glabrata* have increased in frequency with limited antifungal drugs sensitive to them including polyenes, azoles, allylamines, and echinocandins classes due to the evolution of drug resistance rapidly to* Candida* species [10].

Nucleic acid or protein synthesis inhibitor antibiotics like fluoroquinolones and aminoglycosides may have a role in the acceleration of the pharmacological action of antifungal drugs [11, 12].

Moreover, the combination of antibiotics with antifungal drugs, simultaneously or sequentially, is often clinically used, but without a good orientation of the consequences; additionally, the role of combined therapy and the effect of antibiotics upon fungal growth should be assessed and evaluated to prevent unwanted drugs interaction and enforced the synergistic effect of these combined therapies [6, 12–14].

Thus, several studies have investigated the effect of FOA on microbial flora of mouth and especially candidal growth but few of them have investigated the pharmacodynamic effect of antibacterial-antifungal combination on candidal growth.

Based on the above, the objective of this study was to evaluate in vitro pharmacodynamic interactions of antibacterial and antifungals combination by microdilution broth checkerboard techniques by using a modification of the CLSI M27-A3 technique [15] and data can be analyzed by bliss question to determine synergistic and antagonistic interactions.

## 2. Materials and Methods

### 2.1. Patients

74 patients between 11 and 30 years old (males 36% and females 64%) with mean age (19.5 ± 2) years, required treatment with FOA at the Clinics of Orthodontic, Dentistry College, Babylon University. The study was approved by the ethical committee of College of Dentistry, University of Babylon, Hilla, Babylon, Iraq, in accordance with the Declaration of Helsinki.

### 2.2. Inclusion Criteria

Eligible patients should not document chronic systemic diseases, should not be receiving immunosuppressive drugs or antimicrobials in the last month, and should not be using antiseptic rinse prior to sampling, with taking consent from patients for sampling.

### 2.3. Sampling

The study was designed with taking the samples one month after FOA placement. Samples were taken with a sterile swab, which was rubbed rotationally on the oral mucosa and the back of the tongue for each of the patients, in addition to culturing the sterile plastic pads.

### 2.4. Isolation and Identification of Bacteria

The samples were cultured aerobically on blood agar (blood agar base), MacConkey agar, and chocolate agar (DCM, Netherlands). API system for Staph, API 20 Strept for G+ve cocci, and API 20E for G-ve bacilli (bioMérieux's, France) were used.

### 2.5. Isolation and Identification of Candida

The samples were cultured aerobically on Sabouraud Dextrose Agar (Difco Laboratories, Basel, Switzerland) and presumptively identified for each species specific of genus* Candida* in CHROMagar Candida (Hichrome Candida Differential Agar, HiMedia Laboratories, M1297A, India).

### 2.6. MIC (Minimal Inhibitory Concentrations) Testing for Antifungal Drugs

MIC testing was performed according to the NCCLS approved standard M27-A3 [15] for the reference method of broth dilution antifungal susceptibility testing of yeasts. An inoculum suspension was prepared from new cultures with a counting chamber in order to obtain 2 × 10^4^ conidia/mL in 10.4 g/L RPMI 1640 with glutamine without sodium bicarbonate (Sigma-Aldrich, St. Louis, MO) and 0.165 M morpholinepropanesulfonic acid buffer (Invitrogen, Carlsbad, CA), pH 7.0, with 100 mg/L chloramphenicol (Sigma-Aldrich, St. Louis, MO). The reference strain was* Candida albicans* (ATCC 10231) [15].

The MIC of amphotericin B (AMB) (Bristol-Myers Squibb, Princeton, NJ), ketoconazole (KET) (Pfizer Inc., New York, NY, USA), and itraconazole (ITZ) (Pfizer, New York, NY, USA) as antifungal drugs was tested by CLSI method after 24 h of incubation, and fungal growth was assessed spectrophotometrically. For spectrophotometric assessment, fungal growth was measured at 405 nm. The relative optical density (OD) was 405 for each well and drug concentration, in relation to the control well.

All stock antimicrobial solutions were stored in one-time-use aliquots at −70°C. The tests were carried out in duplicate in sterile plastic microplates (TPP Zellkultur Test Plate 96F, Switzerland) with MICs of reference strain being 0.25–1.0 mg/L for AMB, 1.0–2.0 mg/L for KET, and 0.5–1.0 mg/L for ITZ.

### 2.7. The Drugs Combination

The combination screens were performed in 96-well plates. Each well of the microplate received an increasing concentration with maximum concentration (*C*_max_) ranging from 0.015 to 32 mg/L for antifungal agents. Every well had 100-*μ*L of RPMI with inocula and 100-*μ*L of antifungal agents without antibiotics or 50 *μ*L of antifungal agents and 50 *μ*L of antibiotics in other columns of the microplates with 7 concentrations (*C*_max_) from antibiotics ciprofloxacin (CIP), doxycycline (DOX), and metronidazole (MET) (Sigma-Aldrich, St. Louis, MO). The serial dilutions were performed to achieve maximal dilutions.

All the assays were carried out in duplicate. The treated microplates were incubated at 35°C for 24 h. The results were evaluated by optical density of spectrophotometer as relative values at 405 nm [16].

### 2.8. Bliss Independence Interaction Analysis

Bliss independence is described by the equation *I*_IND_ = *I*_*A*_ + *I*_*B*_ − *I*_*A*_ × *I*_*B*_ for a certain combination of (*x*) concentration of drug *A* and (*y*) concentration of drug *B* where *I*_*A*_ is antifungal at (*x*) concentration alone, *I*_*B*_ are antibiotics at (*y*) concentration alone, and *I*_IND_ is expected fungal growth with antibiotic as 100% and antifungal combination as (*x*) concentration of antifungal with (*y*) concentration of antibiotics. Therefore, fungal growth with antibiotic or antifungal (*I*) is equal to 1 − *E*.

Bliss equation can be transformed to *E*_IND_ = *E*_*A*_ × *E*_*B*_, where *E*_*A*_ and *E*_*B*_ are difference in fungal growth with antifungal and antibiotic, respectively. This difference is expected values (*E*_IND_) and the experimentally observed values (*E*_OBS_); thus Δ*I* = *E*_IND_ − *E*_OBS_. If Δ*E* is >0 (*E*_OBS_ < *E*_IND_), Bliss synergy is concluded for that particular combination. If Δ*E* is <0 (*E*_OBS_ > *E*_IND_), hence, Bliss antagonism is concluded for that particular combination. For each combination between antifungal and antibiotic drugs, its statistical significance was assessed by Student's *t*-test [17, 18].

The statistical significance was *p* value less than 0.05 and all statistical analysis was performed with the software Prism 5.01 (GraphPad Inc., La Jolla, CA).

## 3. Results and Discussion

In our study, the prevalence of cariogenic candida in FOA patients was about 24% from collected samples, which was in accordance with previous studies that showed about 30% [2, 19, 20]; furthermore, with all candida isolates being associated with bacterial growth (one or more bacterial isolates) (Table 1) [6], this superadded infection may exert an effect on the virulence of candida. Some studies showed that once the microorganisms (bacteria and fungi) are established in the plaque, they do not inhibit each other, but rather they seem to exert a synergistic effect for caries formation [6, 19]. Our findings supported the previous results that revealed* S. viridans* bacteria as an abundant oral streptococcal infection associated with candida in patients with FOA [6, 21, 22].

### 3.1. In Vitro MICs

In vitro studies for MICs of AMB, KET, and ITR against each candida isolate in addition to associated bacterial pathogens for each isolate were shown in Table 1 while MICs for three antibiotics were inactive for candida isolates. The mean of MICs (mMICs) for AMB was 0.5 mg/L, for KET was 2.0 mg/L, and for ITR was 1.0 mg/L (Table 1).

The results in checkerboard data with intermediate growth could be undetected visually in single-drug experiments by using twofold dilutions. For example, an MIC of 2 mg/L in a twofold dilution scheme may correspond to any concentration between 1 and 2 mg/L [23–26]; moreover, in vivo study showed that the two microorganisms represent a triggering or supporting factor for the periodontitis development in FOA patients [27].

### 3.2. Pharmacodynamic Effects of Combinations

**Figure 1:**
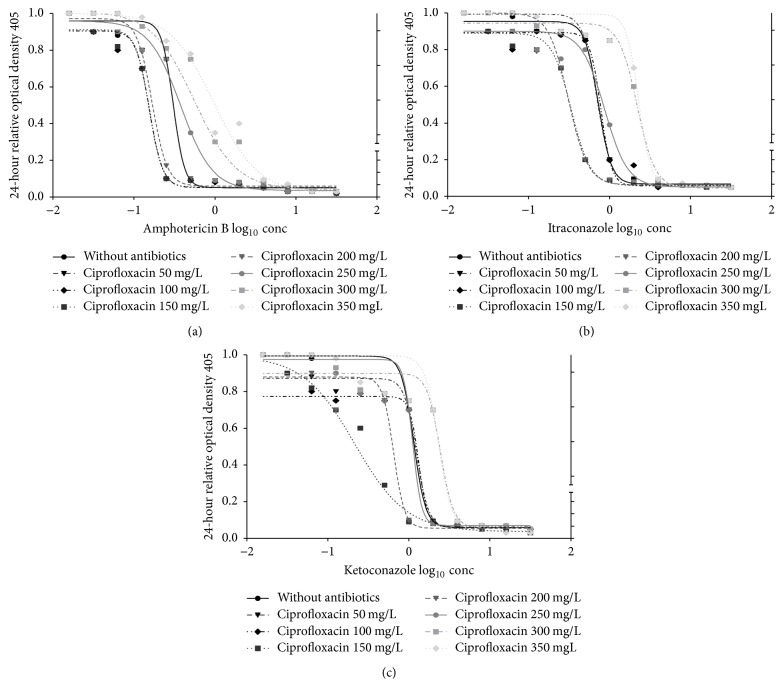
In vitro pharmacodynamic effects of ciprofloxacin (mg/L) combined with amphotericin B (mg/L); ketoconazole (mg/L); itraconazole (mg/L) as log_10_ concentration on candida isolated from patients with fixed orthodontic appliance.

**Figure 2:**
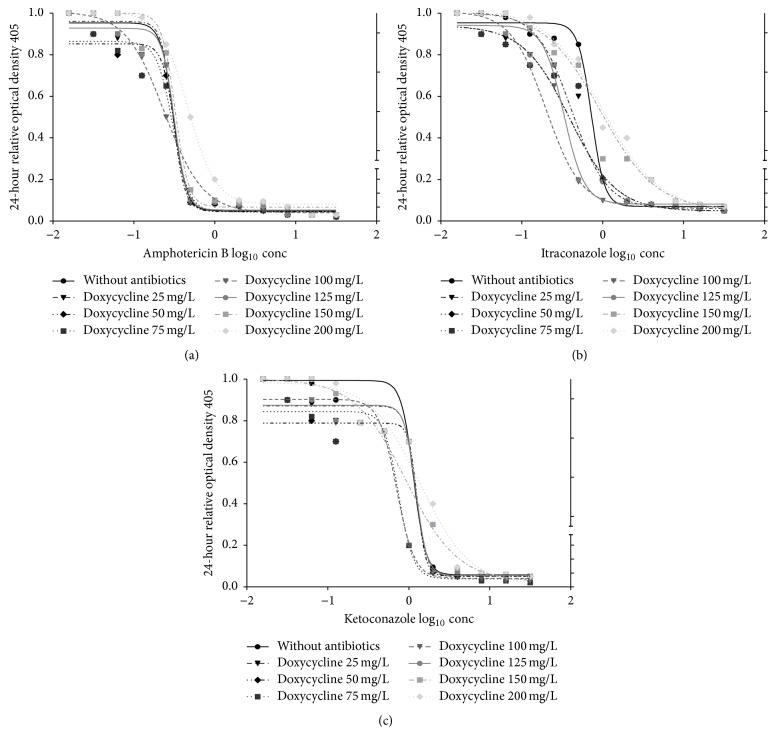
In vitro pharmacodynamic effects of doxycycline (mg/L) combined with amphotericin B (mg/L); ketoconazole (mg/L); itraconazole (mg/L) as log_10_ concentration on candida isolated from patients with fixed orthodontic appliance.

**Figure 3:**
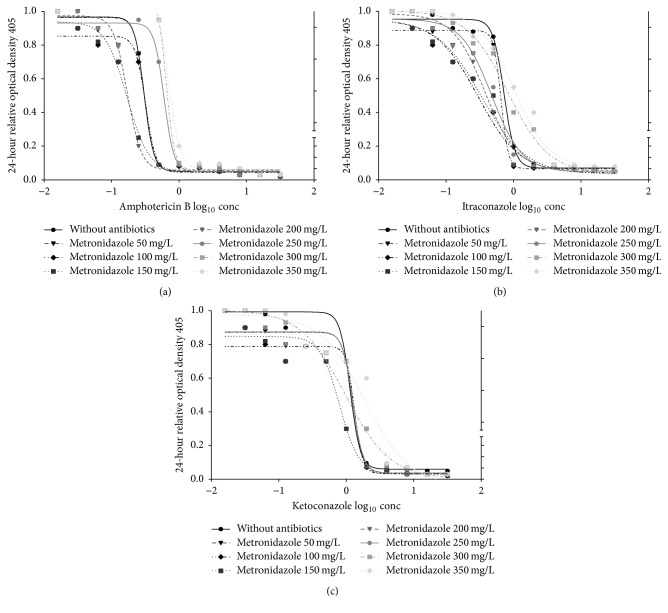
In vitro pharmacodynamic effects of metronidazole (mg/L) combined with amphotericin B (mg/L); ketoconazole (mg/L); itraconazole (mg/L) as log_10_ concentration on candida isolated from patients with fixed orthodontic appliance.

The mMICs for AMB was decreased from 0.5 to 0.25 mg/L in the presence of CIP with *C*_max_ from 100 to 200 mg/L (Figure 1) and of MET with *C*_max_ from 150 to 200 mg/L (Figure 3) while there was no change of mMICs with DOX (Figure 2); moreover, mMICs was increased to 1.0 and 2.0 mg/L with CIP at *C*_max_ between 300 and 350 mg/L (Figure 1), with DOX at 200 mg/L *C*_max_ (Figure 2), and with MET at *C*_max_ between 300 and 350 mg/L (Figure 3).

The mMICs of KET also decreased from 2.0 to 1.0 mg/L in the presence of CIP with *C*_max_ of 150 to 200 mg/L (Figure 1), of DOX with *C*_max_ from 75 to 100 (Figure 2), and of MET at 150 mg/L *C*_max_ (Figure 3) only; furthermore, mMICs was increased to 4.0 and 8.0 mg/L with CIP at *C*_max_ between 300 and 350 mg/L (Figure 1), with DOX at *C*_max_ between 150 and 200 mg/L (Figure 2), and with MET at *C*_max_ between 250 and 350 mg/L (Figure 3).

Regarding the mMICs of ITR, it was decreased from 1.0 to 0.5 mg/L in the presence of CIP with *C*_max_ from 150 to 200 mg/L (Figure 1) and of DOX with *C*_max_ from 75 to 100 (Figure 2) while there was no change of mMICs with MET (Figure 3); in addition to that, mMICs was increased to 2.0 and 4.0 mg/L with CIP at *C*_max_ between 300 and 350 mg/L (Figure 1), with DOX at *C*_max_ between 125 and 200 mg/L (Figure 2), and with MET at *C*_max_ between 250 and 350 mg/L (Figure 3).

Drugs' combinations were presented as two-drug combinations of antifungal and antibacterial drugs with different modes of action. Some studies showed that the growth impairment of candida appeared to be the sum of individual contributions of either chemical to the cells' fitness, rather than a synergistic effect [13, 28, 29].

Regarding DOX, antimicrobial agent that prevents bacterial protein synthesis potentiates the antifungal activity of azole group against candida infection (dosage-dependent); moreover, it converts the action of this antifungal group from fungistatic to fungicidal and prevents the onset of drug resistance later. Additionally, DOX appears to have a major impact on prevention of azole tolerance [13].

### 3.3. Analysis of Pharmacodynamics Effect between Antibiotics and Antifungals according to Bliss Independence Analysis

According to bliss independence interaction analysis on clinical candidal isolates, the effects of antibacterial-antifungal combination were demonstrated by Δ*E* values where the MIC with antibacterial is lesser than without it (*E*_OBS_ < *E*_IND_).

According to these data, the best combination of AMB was with CIP where Δ*E* was 55.14 while with MET, Δ*E* = 40.95, and with DOX, Δ*E* = 40.77 (*p* < 0.001) at *C*_max_ for AMB = 0.25 mg/L.

Regarding KET, the best synergism was with both DOX and CIP where Δ*E* were 42.11 and 41.23 (*p* < 0.001) at *C*_max_ for DOX =100 mg/L and CIP = 150 mg/L, respectively, while with MET, Δ*E* = 15.15 (*p* < 0.05) at *C*_max_ = 150 mg/L, *C*_max_ for KET = 1.0 mg/L.

**Figure 4:**
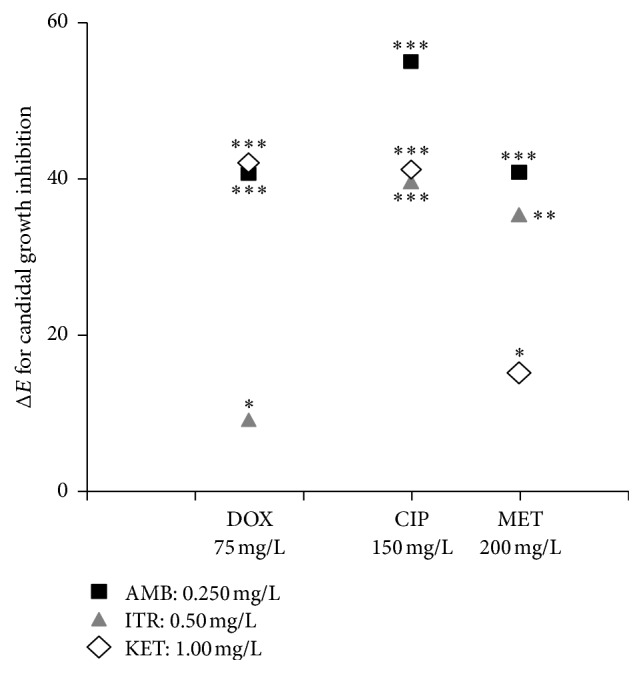
The synergistic effects of antibiotics-antifungal combination according to bliss independence interaction analysis on clinical candida isolated from patients with fixed orthodontic appliance. AMB: amphotericin B (mg/L); KET: ketoconazole (mg/L); ITR: itraconazole (mg/L), CIP: ciprofloxacin, DOX: doxycycline, MET: metronidazole. The number of asterisks (*∗*) corresponds to the level of the statistical significance (^*∗*^*P* < 0.05, ^*∗∗*^*P* < 0.01, and ^*∗∗∗*^*P* < 0.001).

Furthermore, ITR data showed that the best synergism was with CIP where Δ*E* was 39.67 (*p* < 0.001) and with MET where Δ*E* was 35.45 (*p* < 0.01) while with DOX, Δ*E* = 9.12 (*p* < 0.05) at *C*_max_ for ITR = 0.5 mg/L (Figure 4).

On the other hand, Δ*E* for other concentrations were lower than 1.0; that means there was no synergism while Δ*E* values decreased to < −19.50 for CIP *C*_max_ > 300 mg/L, <−13.85 for DOX *C*_max_ > 150 mg/L, and < −21.33 for MET *C*_max_ > 300 mg/L (*p* < 0.001).

The results were evaluated by bliss interaction analysis in order to determine the synergy and antagonism.

There are many in vitro studies exploring the interactions between antifungal compounds and some antibacterial agents depending on use of different agents with certain concentrations. In our study, serial concentrations for different agents have been chosen to evaluate the interaction more deeply and specifically. Aminoglycosides exhibit no antifungal activity on their own, but with amphotericin B they appear to facilitate the drug's entry into the fungal cell, allowing it to inhibit DNA transcription. Indeed, synergy has been found for amphotericin B plus aminoglycosides against isolates of* Candida* spp.,* Aspergillus* spp., and* Fusarium* species [30, 31]. Aminoglycosides also enhance the effects in vitro of azole agents [31, 32]. Several studies have also shown synergy between antifungal agents and the fluoroquinolones such as ciprofloxacin, levofloxacin, and ofloxacin and the macrolides against some fungal species [33–36].

In contrast to in vivo experimental models that may allow clinical effectiveness to be predicted and mimicking to humans infection [37, 38], the in vitro susceptibility testing determines the inherent susceptibilities of organisms to antimicrobial agents which are considered more reliable for ascertaining whether an antimicrobial agent is suitable for treating a human infection and assessing combinations of antifungal-antibacterial agents. Many studies revealed low statistical power to detect significant differences in efficacy of different therapies and raise doubts about the validity of their results for application of antifungal agents that are recommended for clinical use. The inhibition of fungal DNA or protein synthesis might represent a new target for future antifungal agents. The role of DNA or protein synthesis inhibitor antibiotics in combination with antifungal agents as a new therapeutic strategy against candida had been assessed in many studies but not improved yet. On the other hand, the synergy of pharmacokinetic data for these combinations had increased the fungicidal effect of antifungal compounds [31, 36, 39–41].

In conclusion, this study uncovers the optimum antifungal-antibacterial combination therapy against cariogenic candida in dose specific manner by usage of low therapeutic concentrations in patients with FOA. The best synergism was between AMB and CIP and the lowest synergism was between ITR and DOX at low therapeutic concentrations while the antagonistic interactions were detected at highest therapeutic concentrations of antibacterial agents.

## Figures and Tables

**Table 1:** Pharmacodynamic data of candida isolated from patients with fixed orthodontic appliance. MIC: minimal inhibitory concentration (mg/L); AMB: amphotericin B; KET: ketoconazole; ITR: itraconazole.

Candidal isolates	Associated bacteria	MIC for AMB	MIC for KET	MIC for ITR
*C. albicans (1)*	*S. viridans*	0.5	1.0	1.0
*C. albicans (2)*	*S. viridans*	0.5	1.0	1.0
*C. albicans (3)*	*S. viridans*	0.5	1.0	1.0
*C. albicans (4)*	*S. viridans*	0.5	1.0	1.0
*C. albicans (5)*	*P. aeruginosa*, *S. viridans*	0.5	2.0	2.0
*C. albicans (6)*	*P. aeruginosa*	0.5	2.0	1.0
*C. albicans (7)*	*P. aeruginosa*	0.5	2.0	1.0
*C. albicans (8)*	*E. coli*, *S. viridans*	0.5	2.0	2.0
*C. albicans (9)*	*E. coli*	0.5	1.0	1.0
*C. albicans (10)*	*E. coli*	0.5	1.0	2.0
*C. dubliniensis (1)*	*S. viridans*	0.5	1.0	1.0
*C. dubliniensis (2)*	*S. viridans*	0.5	2.0	1.0
*C. dubliniensis (3)*	*E. coli*	0.5	2.0	2.0
*C. parapsilosis (1)*	*S. viridans*	0.5	1.0	1.0
*C. parapsilosis (2)*	*S. aureus, S. viridans*	1.0	1.0	1.0
*C. krusei*	*P. aeruginosa*	0.5	4.0	2.0
*C. glabrata*	*S. viridans*	0.25	1.0	1.0
*C. tropicalis *	*S. viridans*	0.25	1.0	1.0
